# An open and lite technoeconomic dataset and assumptions for open energy, emissions and mitigation modeling in the Republic of South Africa

**DOI:** 10.1016/j.dib.2024.110999

**Published:** 2024-10-05

**Authors:** L. Dixon, B. Mosongo, A. Marquard, N. Nosrati-Ghods, S. De Kock, U. Gogela, F.A. Plazas-Niño, M. Howells

**Affiliations:** a Department of Geography, STEER Centre, Loughborough University, Loughborough, United Kingdom; b Centre for Environmental Policy, Imperial College London, London, United Kingdom; c Energy Systems Research Group, University of Cape Town, Cape Town, South Africa

**Keywords:** Energy modeling, Energy transition, Energy planning, OSeMOSYS, Energy policy

## Abstract

This article provides a large-scale dataset for South African energy system modeling intended to aid long-term energy planning. The dataset, which is accessible on Zenodo, contains comprehensive data on installed capacity, resource potentials, historical and projected electricity production and consumption, capacity factors, and capital, fixed, and variable costs. The data was rigorously collected from national institutions, international organizations, and scholarly journals and organized into an easy-to-use Excel workbook. This dataset is compatible with the open-source energy modeling system (OSeMOSYS) framework but can also be used in other energy modeling applications [1]. Following the U4RIA (ubuntu, retrievability, repeatability, reconstructability, interoperability, and auditability) principles [2], this dataset attempts to improve energy modeling efforts, supporting analysts, policy makers, and the scientific community in creating robust energy strategies. By lowering the complexity and time needed for data collection, this tool

helps South Africa, and other comparable regions achieve more effective and efficient energy planning.

Specifications Table

**Table:** 

Subject	Energy
Specific subject area	Energy System Modeling
Data format	Raw, Processed, Analyzed
Type of data	Tables, Graphs
Data collection	Data was collected from websites, reports, and databases from international organizations and different energy related national institutions in South Africa, as well as from academic articles
Data source location	Raw data sources are listed in the different sections of this article
Data accessibility	With this article and in a repository Repository name: Zenodo Data identification number: 10.5281/zenodo.13124390 Direct URL to data: https://zenodo.org/records/13710940

## 1 Value of the Data

• This dataset can be used to develop energy system models and to investigate renewable energy transition pathways in South Africa. This combination of different hypotheses and scenario frameworks can provide useful insights for policymakers.
• The data addresses issues related to a difficult and time-consuming data collection process by being open-source, comprehensive, and accessible.
• Analysts, decision makers, and the scientific community can use the dataset, and the methodologies provided to undertake energy studies in South Africa and other similar countries. Furthermore, governments may better define the role of public financing, increase access to global climate funding, and strategically allocate financial resources for implementation based on the results of energy system analysis [3].

## 2 Background

Effective long-term energy planning hinges on accurate and accessible data to enable robust energy systems modeling. However, national-scale modeling projects frequently encounter major obstacles, particularly in regions like South Africa, where data quality, availability and accessibility issues are prevalent [4,5]. Historically, energy systems modelling has been time-intensive and required advanced pre-existing knowledge, experience, and skill [6]. Nevertheless, government agencies and academic institutions in South Africa have been involved in various modelling efforts.

Since 2010 the Department of Mineral Resources and Energy has been developing its Integrated Resource Plan (IRP) using PLEXOS. The IRP outlines a long-term strategy for diversifying South Africa's energy mix, leveraging its regional position as an energy export leader and the second highest consumer of electricity per capita [7].

The Energy Systems Research Group (ESRG) at the University of Cape Town, established in 2002 and restructured in 2019/20, has played a key role in modelling energy and economic systems. ESRG manages SATIM (South African TIMES model), the country's only comprehensive energy sector model, which has been central to studies like Bruno et al. (2018) for least-cost optimization in renewable energy transitions [8]. SATIMGE, a hybrid of SATIM and the eSAGE model, enables detailed analysis of both energy systems and economic detail for assessing the impact of changes in the energy system on various sectors, markets, and agents [9].

While progress in energy systems modelling is evident, the complexity associated with these models can limit their accessibility. Additionally, the modelling and cost assumptions behind the IRP have been heavily criticized for their lack of transparency [10]. Recent global studies, including Cannone et al. (2023) and Brinkerink et al. (2021), have proposed solutions to enhance accessibility and improve energy modeling practices [4,5]. Addressing these challenges, this work provides a publicly available dataset specifically designed for long-term energy planning in South Africa. Developed as part of the Energy Modeling Platform for Africa 2024, it builds on the Starter Data Kit for South Africa [11], incorporating up-to-date information on installed capacity, demand projections, and technology costs.

With the intent to improve access and encourage further model development, this basic dataset's primary objective is to provide a localized resource for policymakers and researchers. The technoeconomic dataset aligns with the U4RIA principles of Ubuntu, Retrievability, Repeatability, Reconstructability, Interoperability, and Auditability [2]. Continually updating the input data is crucial for the modeling process, as the initial characteristics of an energy system impact the model's evolution and introduce significant inertia, thereby influencing the results. This dataset was constructed for input into an OSeMOSYS model and can be readily employed and built upon in this context or in similar long term energy modeling frameworks.

## 3 Data Description

This paper presents a dataset designed for energy modeling of long-term power sector transition planning in South Africa using OSeMOSYS. It is important to note that the data provided in this document exists independently of the tool. The dataset can be accessed on the Zenodo repository through the following link: https://zenodo.org/records/13710940.

The data is sourced from publicly accessible existing model databases, such as national institutions in South Africa, and includes information on capital, fixed and variable costs, capacity factors, historical and projected electricity production, and demand, installed capacity, and resource potential.

The data is organized into an Excel Workbook consisting of multiple tabs, a single open collection in a user-friendly format. The data is ready for analysts to use, regardless of the energy modeling software they choose, even though it was designed to be fully compatible with clicSAND software [12] for the Open-Source energy modeling system (OSeMOSYS) [1].

### 3.1 Residual Capacity

**Fig. 1:**
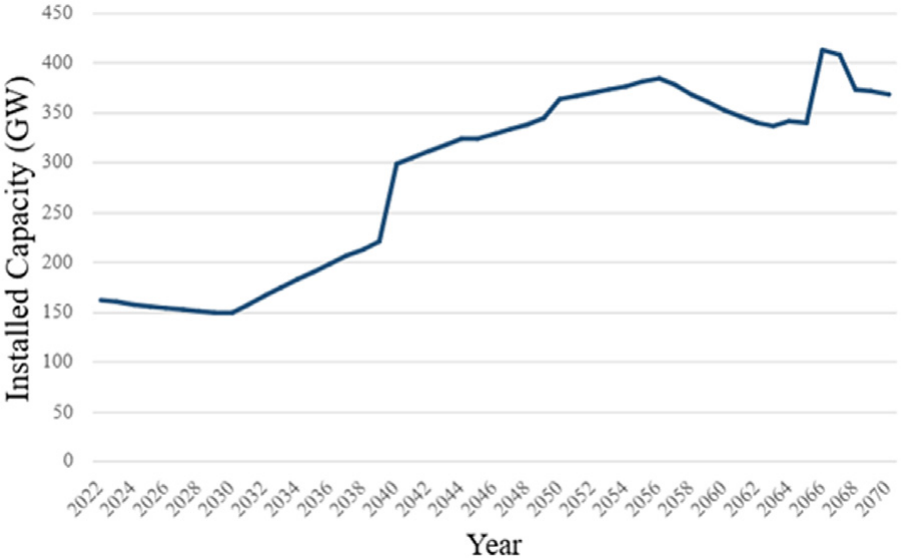
Baseline capacity of the OSeMOSYS model.

Table 1 shows an excerpt of the installed capacity data for key years by Technology. Fig. 1 represents the baseline capacity model before optimization. The residual capacity data represent the existing stock of power plants in South Africa from 2015 to 2050, considering grid-connected capacity as well as off-grid and approved capacity extensions. The complete dataset is accessible in the Excel Sheet ‘Residual Capacity’ in the repository.

**Table 1:** Installed capacity by power generation technologies in key years.

Technology	2022	2030	2040	2050
Biomass Power Plant	0.0250	0.0166	0.00993	0.00594
Coal Power Plant	39.8	30.3	21.6	15.4
Gas Power Plant (OCGT)	3.40	2.26	1.35	0.809
Solar PV (Utility)	2.38	1.58	0.945	0.566
Large Hydropower Plant*	3.32	2.21	1.32	0.791
Onshore Wind	3.40	2.26	1.35	0.809
Nuclear	1.90	1.26	0.755	0.452
Concentrated Solar Plant (CSP)	0.500	0.332	0.199	0.119
Distributed Solar PV w/Storage	1.63	1.08	0.649	0.389
Transmission	32.9	33.9	33.9	33.9
Distribution	29.4	33.0	34.7	49.1

⁎ Utility and Pump Storage >100 MW

### 3.2 Historical Electricity Generation

**Fig. 2:**
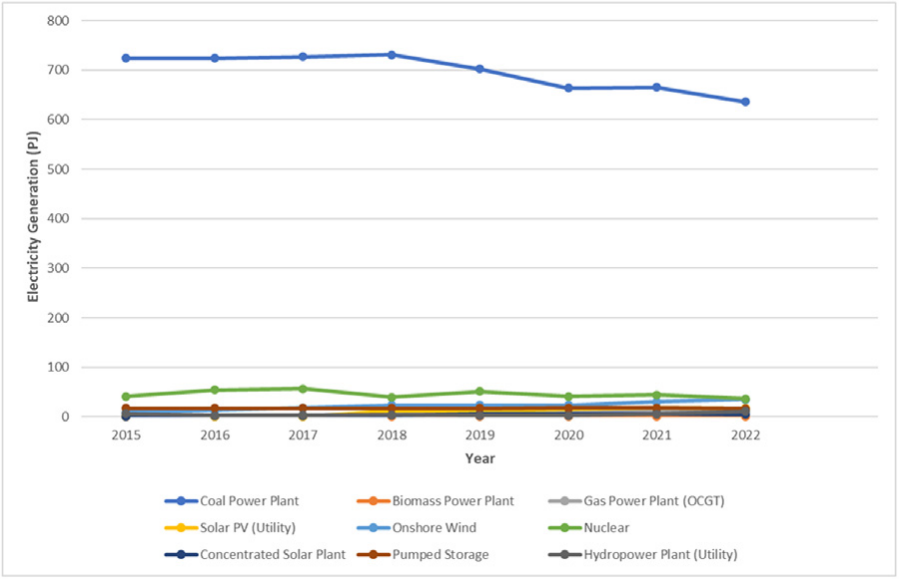
Historical generation by technology 2015-2022.

The data for 2015-2022 for each plant type are summarized in Table 2 and represented graphically in Fig. 2. The generation data represents the power generation in petajoules in historical years from each of the different technologies represented in the model. The full data can be found in the Excel sheet ‘Generation’ in the Zenodo repository.

**Table 2:** Historical generation by power generation technology type (PJ).

Plant Type	2015	2016	2017	2018	2019	2020	2021	2022
Biomass Power Plant	0.885	0.885	0.107	0.670	0.670	0.670	0.670	0.670
Coal Power Plant	724	724	272	731	702	664	665	636
Gas Power Plant (OCGT)	0.000	0.108	0.432	3.600	4.90	6.84	11.52	13.0
Solar PV (Utility)	0.571	0.571	0.571	11.9	11.9	14.8	18.4	17.3
Onshore Wind	9.00	13.3	18.0	23.4	23.8	23.8	30.2	34.9
Nuclear Power Plant	41.3	54.0	56.7	39.6	57.2	41.4	43.9	36.4
Concentrated Solar Plant	0.000	1.80	2.52	3.60	5.76	5.76	6.12	5.04
Pumped Storage	17.4⁎⁎	17.4⁎⁎	17.4⁎⁎	17.2	17.4⁎⁎	13.4	17.6	16.2
*Utility Hydropower Plant	6.61	2.35	3.04	4.39	2.82	2.88	5.76	11.2

⁎ Pumped storage and utility together make up the value used for large hydropower (>100 MW)

⁎⁎ Data points for 2015, 2016, 2017, and 2019 were assumed based on related installed capacity and average capacity factor.

### 3.3 Electricity Demand

**Fig. 3:**
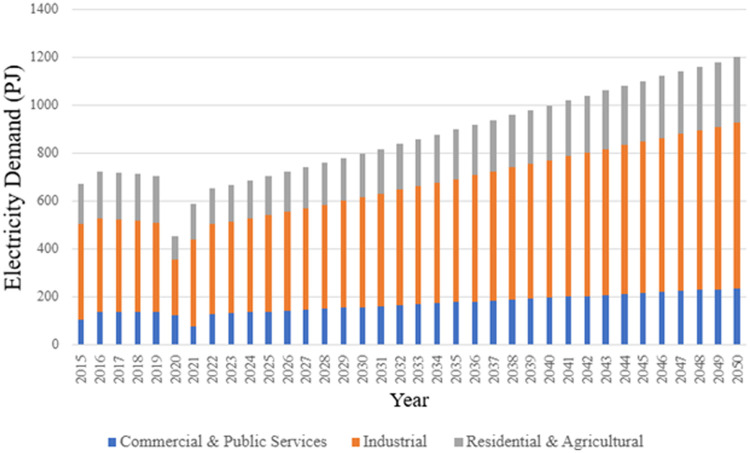
Projected electricity demand by sector 2015-2050.

Table 3 presents an excerpt of the demand data by sector for key years, and Fig. 3 graphically displays the projected electricity demand data, highlighting a decline in electricity demand during the COVID-19 pandemic. The electricity demand data represents both historical and projected electricity demand for 2015–2050 from the industrial, residential, and agricultural, and commercial sectors. The complete dataset is accessible in the Excel Sheet ‘Demand’ in the Zenodo repository.

**Table 3:** Total electricity demand of key years (PJ).

Demand	2023	2030	2040	2050
Industrial	383	458	574	689
Residential and agricultural	153	182	229	275
Commercial	130	155	194	233
Total Electricity Demand	665	796	996	1200

### 3.4 Electricity Imports and Exports

Table 4 outlines data in key years for the total electricity generation imported and exported per year in PJ from the South African energy system. Electricity imports remain constant until 2025 and then decline to zero, aligning with the strategy of achieving sovereignty. Moreover, electricity exports will remain steady until 2030, followed by an annual increase of 0.5%. The complete dataset is accessible in the Excel Sheet ‘Imports & Exports’ in the Zenodo repository.

**Table 4:** Total electricity generation imported and exported (PJ).

Technology	2015	2023	2030	2040	2050
Electricity Imports low bound	47.0	38.9	19.4	0	0
Electricity Exports low bound	52.6	45.4	45.4	47.7	50.2
Electricity Imports high bound	47.0	30.9	11.5	0	0
Electricity Exports high bound	52.6	45.4	45.4	47.7	50.1

### 3.5 Capacity Factors

The capacity factor represents the overall utilization ratio of a power generator through the energy generated over a given period compared to its full capacity. The average capacity factor collected for each power technology is available in Table 5 and in the full dataset for eight timeslices is available in the Excel file of the repository under the sheet ‘Capacity Factor & Lifetime’.

**Table 5:** Average capacity factor of power generation technologies.

Technology	Capacity Factor
Biomass Power Plant	0.850
Coal Power Plant	0.570
Geothermal Power Plant	0.794
Light Fuel Oil Power Plant	0.800
Oil Fire Gas Turbine (SCGT)	0.800
Gas Power Plant (CCGT)	0.500
Gas Power Plant (OCGT)	0.100
Solar PV (Utility)	0.155315
Concentrated Solar Plant (CSP)	0.452
CSP w/Storage	0.400
Hydropower Plants*	0.54261
Onshore Wind	0.360
Nuclear Power Plant	0.850
Onshore Wind w/Storage	0.0777
Light Fuel Generator	0.300
Distributed Solar PV w/Storage	0.181201

⁎ Values applicable to small, medium, large, and off-grid hydropower plant facilities

### 3.6 Resource Potentials and Operating Life for Power Generation Technologies

Operating Life, outlined in Table 6, represents the useful lifetime of a technology, expressed in years and in the Excel file of the repository under the sheet ‘Capacity Factor & Lifetime’. Summarized data on resource and technical potential is presented in Table 7, Table 8 below and can be found in the Excel sheet ‘Resource Potential’ in the Zenodo repository. The data for the resource potential of fossil fuels represents the total energy (PJ) that can be generated from reserves in the country. Thus, the values for coal, oil, natural gas, and uranium represent the maximum total energy that can be generated from South Africa's domestic fuel reserves (PJ). The technical potential for renewables represents the technically feasible maximum power generation capacity in GW each year.

**Table 6:** Operating life of power generating technologies.

Technology	Years
Biomass Power Plant	30.0
Coal Power Plant	35.0
Gas Power Plant (CCGT)	30.0
Solar PV (Utility)	24.0
CSP w/Storage	30.0
Onshore Wind	25.0
Nuclear Power Plant	50.0
Light Fuel Generator	10.0
Distributed Solar PV w/Storage	24.0

**Table 7:** Resource potential for fossil fuel generation (PJ).

Technology	Potential
Oil	8522.04
Coal	0
Natural Gas	509.5
Uranium	8522

**Table 8:** Resource potential for renewable energy generation (GW).

Technology	Potential
Large Hydropower Plant (>100MW)	4.04
Medium Hydropower Plant (10-100MW)	0.455
Small Hydropower Plant (<10MW)	0.180
Off-grid Hydropower	0.020
Geothermal Power Plant	0.450
Offshore Wind	0.000*

⁎ Years 2015-2024

### 2.7 Capital, Fixed, and Variable Costs

An excerpt of data for Capital, Fixed and Variable costs is shown in Table 9, Table 10, Table 11 below. The capital cost represents the overnight cost for a technology from the year it becomes commercially available to the end of the modeling period. The fixed cost is the operation and maintenance cost independent of the production output of the technology per year. The variable costs represent the associated fuel costs or other various O&M expenses. All the data is available in the ‘Costs’ tab of the Excel repository.

**Table 9:** Capital cost of key years ($/kW).

Technology	2020	2025	2030	2035	2040	2045	2050
Biomass Power Plant	5390	4710	4490	4340	4190	4040	3870
Coal Power Plant	3300	3300	3300	3300	3300	3300	3300
Geothermal Power Plant	6750	6880	6710	6550	6383	6230	6070
Oil Fire Gas Turbine (SCGT)	1120	1090	1050	1010	961	917	872
Gas Power Plant (CCGT)	1250	1220	1180	1140	1100	1060	1020
Solar PV (Utility)	987	898	809	766	721	677	632
Onshore Wind	987	898	809	766	721	677	632
Solar PV (Utility) w/Storage	2100	2260	1970	1840	1760	1620	1500
Onshore Wind w/Storage	2330	2580	2250	2120	2010	1900	1810
Distributed Solar PV w/Storage	1410	1332	1258	1184	1110	1036	962
Concentrated Solar Plant	6920	6940	6960	6960	6960	6960	6960

**Table 10:** Fixed cost of key years ($/kW/Year).

Technology	2020	2025	2030	2035	2040	2045	2050
Biomass Power Plant	157	157	157	157	157	157	157
Coal Power Plant	77.5	77.5	77.5	77.5	77.5	77.5	77.5
Geothermal Power Plant	114	114	114	114	114	114	114
Oil Fire Gas Turbine (SCGT)	24.0	23.6	22.9	22.3	21.6	20.9	20.3
Gas Power Plant (CCGT)	31.1	30.6	29.7	28.8	27.9	27.0	26.1
Solar PV (Utility)	23.7	23.7	23.7	23.7	23.7	23.7	23.7
Onshore Wind	50.8	50.8	50.8	50.8	50.8	50.8	50.8
Solar PV (Utility) w/Storage	70.7	68.3	65.7	63.0	62.1	61.1	60.2
Onshore Wind w/Storage	36.5	35.9	35.2	34.6	34.1	33.5	33.0
Distributed Solar PV w/Storage	38.3	38.3	38.3	38.3	38.3	38.3	38.3
CSP w/Storage	78.9	78.9	78.9	78.9	78.9	78.9	78.9
Nuclear Power Plant	119	119	119	119	119	119	119

**Table 11:** Variable cost of key years ($/kWh).

Technology	Cost
Biomass Power Plant	5.04
Coal Power Plant	0.00671
Solar PV (Utility)	5.5E-6
CSP w/Storage	5.5E-5
Onshore Wind	5.5E-6
Nuclear Power Plant	3.13

## 4 Experimental Design, Materials, and Methods

The dataset was compiled through a comprehensive literature review. The data was gathered from national and international databases, scholarly articles, and reports from institutions like ESKOM, DMRE, and CSIR. The main data sources included the Eskom Data Portal, Integrated Resource Plan (IRP), and Renewable Energy Data and Information Service. Data was then processed according to the modelling requirements. Collected data from various sources were normalized to a common unit, (e.g., PJ for energy data). Additional standardization protocols were undertaken, including adjustments to account for discrepancies in reporting periods and unavailability of data. Methodologies used for processing and standardization of data are detailed in the following sections. Following collection and processing, datapoints were then validated and error checked using methods described in Section 4.8.

### 4.1 Residual Capacity

The data on the existing capacity for coal, gas power plants, solar power, CSP with storage, large hydropower, onshore wind, and nuclear power were sourced from CSIR statistics found in the Eskom Data Portal and the 2019 and 2023 IRP [[10], [13], [14]]. The data on the existing capacity for biomass, hydropower plants and onshore wind was taken from the renewable energy data and information [15]. The large hydropower technology represents both pumped storage and utility hydropower, the capacity of these technologies remains constant until the end of the modeling horizon. o data was found on the installed capacity of biomass in the years 2018 to 2022, so the value was assumed at 0.025 – the required capacity to meet historical generation according to Eq. (1). In addition, 720 MW of coal, 419 MW of wind and 75 MW of solar PV became operational in 2022. Planned capacity extensions for power generation up to 2030 were taken from the 2023 IRP [10]. The residual capacities of the transmission and distribution systems were assumed to remain constant from 2022 onward. Capacity into the future for power technologies were extrapolated using the commission date and average operational lifecycle of each power plant type, available in sheet ‘Capacity Factor & Lifetime’ in the Zenodo repository [11,16]. The decline rate of existing capacity was set at 95% for all technologies, except for Coal Power Plant, which had a decline rate of 96.5%. This was done to phase out available technologies and bring their capacity close to zero by 2070. In this case, the model will select the best technology based on cost, capacity, and efficiency.

### 4.2 Historical Electricity Generation

Historical generation data were updated based on generation per plant type found in the CSIR Statistics of utility scale power generation in South Africa 2015-2022 [13], in addition to coal generation data from the DMRE and gas generation data from the ember climate [17,18]. Pumped storage generation data were unavailable for 2015, 2016, 2017, and 2019. Therefore, the values for these years were estimated based on the related installed capacity and average capacity factor using Eq. (1).

(1) $$\begin{array}{ccc} Estimated\;power\;generation\;\left[PJ\right] & = & Residual\;Capacity\;\left[GW\right]\;*\;Average\;Capacity\;Factor\\ & & *\;31.536\;\left[PJGW\right] \end{array}$$

### 4.3 Electricity Demand

The projections for corresponding final energy services were sectioned into commercial, industrial, and residential categories based on the breakdown of sector demand [18,19]. The final energy demand was adjusted from the historical total annual demand provided by the DMRE [17] with Eq. (2). Demand beyond the historical data was projected using a basic linear correlation approach; the growth rate of demand was 2.6% in 2024 and decreased steadily to 1% by 2070 [16]. There is a possibility for improving the projections by considering more detailed population factors in future work. Region-specific efficiencies for power transmission and distribution were also calculated based on the electricity demand, electricity imports, and electricity exports from the DMRE [17].

(2) FinalDemand[PJ]=∑Energyconsumption[PJ]÷InputActivityRatioTechs

### 4.4 Imports and Exports

Information on historical electricity import and export demand was updated from ESKOM CSIR data [13] as well as from the DMRE website [17] and converted from TJ into PJ. Due to small discrepancies between the data from the two sources, higher and lower estimates were set as upper and lower bounds, respectively, for electricity generation from imports and exports in the model. This demand was then projected into the future considering guidance from [20]. However, electricity imports remain constant until 2025 and then decline to zero as part of the strategy to achieve energy sovereignty. Electricity exports remain constant until 2030 and then increase by 0.5% annually.

### 4.5 Capacity Factor

The capacity factors for fossil fuel, biomass, nuclear and gas technologies were sourced from Meridian Economics [14]. The capacity factors for wind, solar, and hydropower technologies were obtained from Brinkerink, M., Gallachóir, B. & Deane, P 2021 study and the Ninja Renewables database [2,18].

### 4.6 Technical Lifetime, Resource Potential and Renewable Energy Potential

The operational lifetimes for power plants were taken from Meridian Economics [21]. Fossil fuels and nuclear power are finite resources; therefore, it is necessary to update the quantity of available reserves for each fuel, crude oil, natural gas, and uranium. Available data were collected for the year 2015 and converted to PJ units of production, and reserves are typically found in volume/weight for fossil fuels. The sum of annual production in the years up to year 2023 was added to the reserve data to calculate the total reserve for the modeling horizon. It is also important to set limits on renewable energy potential, constraining the maximum available energy per year. The data were collected from ESKOM on potential energy production in PJ [13]. If data are available in capacity units, we can estimate the energy produced using the average capacity factor of the technology following Eq. (3):

(3) $$\begin{array}{cc} Estimated\;energy\;production\;\left(PJ\right) & =Maximum\;Installed\;Capacity\;\left(GW\right)\\ & *\;Average\;Capacity\;Factor*31.536\;\left(PJGW\right) \end{array}$$

### 4.7 Technology Costs

The data on capital, fixed and variable costs for the years 2020 to 2050 came from the Meridian Economics Comparative Analysis of IRP 2023 cost assumptions and associated cost data sheets [10]. A deflator of 0.05497 was applied to convert values ZAR to USD, this was the exchange rate in April 2023 at the time of the report [22].

### 4.8 Data Validation and Error Correction

It is important to validate data estimates by confirming values fall within acceptable ranges. As most data collected is raw data from primary sources, validation involved cross-checking datapoints with independent references. For example, generation figures from ESKOM were compared to DMRE statistics. Demand projections were crosschecked with the demand projections in the SATIM model at the UCT and found to be reasonable. Additionally, data estimates for cost and performance data were compared against benchmarks from the Global and National Energy Systems Techno-Economic (GNESTE) Database [23] to ensure they fall within expected ranges. Cost, capacity factor and operational life data for the power generating technologies included in this dataset fall within global averages available in open-source datasets and therefore are considered reasonable estimates for use in modelling the South African energy system.

## Limitations

Not applicable.

## Ethics Statement

Authors have read and followed the ethical requirements for publication in Data in Brief. This work does not involve studies with animals and humans.

## Funding

This work was funded by the South African-driven Modeling Capacity and Communication (ADMeCC) project and Climate Compatible Growth (CCG) programme. The CCG programme brings together leading research organizations and is led out of the STEER centre, Loughborough University. Both CCG and ADMeCC are funded by the Foreign, Commonwealth and Development Office (FCDO) from the UK government; however, the views expressed herein do not necessarily reflect the UK government's official policies.

## U4RIA Compliance Statement

This work follows the U4RIA guidelines, which provide a set of high-level goals relating to conducting energy system analyses in countries [2]. This research was conducted involving stakeholders in the development of models, assumptions, scenarios and results (Ubuntu/ Community). The authors ensure that all data, source code and results can be easily found, accessed, downloaded, and viewed (retrievability), licensed for reuse (reusability), and that the modelling process can be repeated in an automatic way (repeatability). The authors provide complete metadata for reconstructing the modeling process (reconstructability), ensuring the transfer of data, assumptions and results to other projects, analyses, and models (interoperability), and facilitating peer review through transparency (auditability).

## CRediT authorship contribution statement

**L. Dixon:** Writing – original draft, Writing – review & editing. **B. Mosongo:** Writing – original draft, Writing – review & editing. **A. Marquard:** Supervision. **N. Nosrati-Ghods:** Conceptualization, Methodology, Formal analysis, Investigation, Data curation, Writing – review & editing. **S. De Kock:** Conceptualization, Methodology, Formal analysis, Investigation, Data curation. **U. Gogela:** Conceptualization, Methodology, Formal analysis, Investigation, Data curation. **F.A. Plazas-Niño:** Supervision. **M. Howells:** Supervision.

## Data Availability

ZenodoOpen and Lite Techno-economic Dataset for Long-term Energy Systems Modelling in the Republic of South Africa (Original data).
